# Clinical differentiation of inflammatory bowel disease (IBD) in Latin America and the Caribbean

**DOI:** 10.1097/MD.0000000000028624

**Published:** 2022-01-21

**Authors:** Jesús K. Yamamoto-Furusho, Norma N. Parra-Holguín, Fabián Juliao-Baños, Fabián Puentes, Rocio López, Francisco Bosques-Padilla, Esther A. Torres, Humberto Nieves-Jimenéz, Guillermo R. Veitia-Velásquez, Maria L. Jara-Alba, Sócrates Bautista, Felipe N. Piñol-Jimenez, Pablo Salgado-Rosado, Keyla C. Villa-Ovalles, Yudelka A. Abreu-Martinez, Zunilda Borges, Santiago Davila-Bedoya, Guillermo Otoya-Moreno, Beatriz Iadé-Vergara

**Affiliations:** a Inflammatory Bowel Disease Clinic, Gastroenterology Department, National Institute of Medical Science and Nutrition Salvador Zubirán, Mexico City, Mexico; b Hospital Pablo Tobón Uribe, Medellín, Colombia; c United Surgeons, Manizales, Colombia; d Santa Fe Foundation, Bogotá, Colombia; e Department of Gastroenterology, Hospital Universitario Dr. José Eleuterio González, Universidad Autónoma de Nuevo León, Monterrey, Nuevo León, Mexico; f University of Puerto Rico, San Juan, Puerto Rico; g Gastroenterology Service, Hospital Vargas de Caracas, Caracas, Venezuela; h Hospital Dr. Teodoro Maldonado Carbo - Instituto Ecuatoriano de Seguridad Social (IESS), Guayaquil, Ecuador; i Centros de Diagnóstico y Medicina Avanzada y de Conferencias Médicas y Telemedicina (CEDIMAT) Gastroenterology Center, Santo Domingo, Dominican Republic; j National Center for Minimally Access Surgery, Habana, Cuba; k Regional University Hospital José María Cabral y Baez, Santiago de los Caballeros, Dominican Republic; l Hospital Carlos Andrade Marin, Quito, Ecuador; m Private Practice, Lima, Perú; n Centro de Asistencia del Sindicato Médico del Uruguay (CASMU) Cooperativa de Servicios Médicos (COSEM), Uruguay; o Pan American Crohn's and Colitis Organization (PANCCO), Mexico City, Mexico.

**Keywords:** Crohn disease, epidemiology, inflammatory bowel disease, Latin America, ulcerative colitis

## Abstract

The aim of the present study was to describe the epidemiological and clinical characteristics of inflammatory bowel disease (IBD), including medical and surgical treatments, in several countries in Latin America and the Caribbean.

IBD is recognized as a global health problem because its incidence and prevalence have increased significantly over the last few years.

This multicenter retrospective cohort study included 4714 patients with IBD diagnosed from 9 countries in Latin America and the Caribbean: Colombia, Cuba, Dominican Republic, Ecuador, Mexico, Peru, Puerto Rico, Uruguay, and Venezuela.

Crohn disease (CD) was more frequent in Puerto Rico (71.9%), the Dominican Republic (61.0%), and Peru (53.1%). Ulcerative colitis was more frequent in Colombia (78.6%), Venezuela (78.2%), Mexico (75.5%), Cuba (69.9%), Ecuador (64.1%), and Uruguay (60.9%). The following clinical characteristics were more frequent in the Caribbean: penetrating behavior in CD, steroid dependence, steroid resistance, intolerance to thiopurines, extraintestinal manifestations, surgeries, hospitalizations due to IBD, and family history of IBD. The factors associated with the use of biological therapy were pancolitis in ulcerative colitis, penetrating behavior in CD, steroid resistance and dependence, presence of extraintestinal manifestations, and the need for surgery.

This study from Latin America and the Caribbean demonstrated the different epidemiological and clinical characteristics of IBD.

## 1 Introduction

Inflammatory bowel disease (IBD) includes ulcerative colitis (UC), Crohn disease (CD), and indeterminate colitis (IC). The etiology remains unknown; however, it is a multifactorial condition involving genetic, immunological, and environmental factors.^[1]^ In recent years, numerous epidemiological studies from industrialized countries in Asia and Latin America have reported a sudden increase in the incidence of IBD.^[2,3]^ It is currently recognized as a global health problem, as its incidence and prevalence have increased significantly over the last few years.^[4]^ In contrast to the Western world, a peak in the incidence of IBD in Asia and Latin America has yet to be reached.^[5]^ Countries near the poles have the highest prevalence and incidence compared with countries near the equator, where they present the lowest incidence but have increased recently.^[6]^ Unfortunately, there are limited epidemiological data in most developing countries, and the importance of changing this is that knowledge of the global epidemiology of IBD is fundamental for understanding each aspect of IBD.^[4]^ To provide an overview of the epidemiology of IBD, the incidence and prevalence of IBD are higher in Scandinavian countries, Great Britain, Canada, and the United States than in Central Europe, Africa, and Asia.^[7,8]^ This is consistent with previous studies that reported an increase in the incidence of IBD in regions where it was considered to have low incidence, such as Eastern Europe, Asia, Africa, and Latin America.^[7,9]^ In the United States, it is estimated that approximately 1.4 million people have IBD, with an estimated prevalence of 201 cases per 100,000 for CD and 238 cases per 100,000 for UC.^[8,9]^ Currently, in Europe, the prevalence seems to be higher since it has been estimated at 505 cases of UC per 100,000 inhabitants and 322 cases of CD per 100,000 inhabitants. However, in Asia and the Middle East, it is estimated to be 168 cases per 100,000 for UC and 67.9 cases per 100,000 for CD.^[4]^ In China, the prevalence seems to be increasing, although not as markedly as that in Western countries.^[9]^ In Latin America, there are few CD data; however, for UC, the prevalence in Brazil is 38.2/100,000 cases,^[10]^ in Puerto Rico it was estimated at 12.53/100,000 prevalence, the incidence of 1.96/100,000 in 1996 to 3.32/100,000 in 2000, and for CD it increased from 0.49/100,000 in 1996 to 1.96/100,000 in 2000.^[11]^ In Colombia, from 2001 to 2009, 80.7% had UC, and 15.8% had CD.^[12]^ The incidence of UC reported in Panama was 1.2/100,000 per year, with no reported cases of CD, and in Argentina 2.2/100,000 per year, with only one case identified with CD.^[13]^ In Chile, the estimated prevalence has not been determined, but a descriptive study reported that approximately 71% of IBD cases have UC and 27% have CD.^[14]^ In Mexico, the incidence was reported during the period 2000 to 2017, the incidence of IBD has constantly increased from 0.05 to 0.21 per 100,000 in the last 15 years. The incidence of new cases of IBD has increased significantly in the last 15 years, 5.9 times for IBD, 5.3 times for UC, and 9.5 times for CD. The prevalence rates of IBD, UC, and CD were 1.83, 1.45, and 0.34 cases per 100,000 persons per year.^[15]^ There is a large gap in the knowledge regarding the epidemiological and clinical characteristics of IBD in Latin American and Caribbean countries. Therefore, the aim of the present study was to describe the epidemiological and clinical characteristics, including medical and surgical treatments for IBD, in several countries of Latin America and the Caribbean.

## 2 Material and methods

This multicenter retrospective cohort study included 9 countries from Latin America and the Caribbean: Colombia, Cuba, Dominican Republic, Ecuador, Mexico, Peru, Puerto Rico, Uruguay, and Venezuela, from August 2017 to April 2020. All patients had a confirmed diagnosis of IBD based on the clinical, biochemical, endoscopic, histopathological, and radiological features and had all the data of clinical and social demographics, which first came to medical appointment or were already followed up in any of the centers included. Data collection was performed by filling out an individual electronic collection sheet for each patient by reviewing clinical records, which included several demographic and clinical characteristics of each subtype of IBD. Each participating physician sent the collection sheet to the study coordinating center located in Mexico, where all data were reviewed, approved, and included in an electronic database.

Two study groups were created according to geographic region. Group 1 consisted of Caribbean countries, such as Cuba, Puerto Rico, and the Dominican Republic; Group 2 consisted of Latin American countries, such as Colombia, Ecuador, Mexico, Uruguay, Venezuela, and Peru. All variables considered in the electronic collection sheet were as follows: sex, current age, age at diagnosis of IBD (classified according to the following age groups: <16 years; 17–40 years; and >40 years), disease duration, employment status, family history of immune-mediated diseases, family history of IBD, smoking, appendectomy, tonsillectomy, atopic dermatitis, allergic rhinitis, asthma, and presence of extraintestinal manifestations (EIMs) such as arthralgia, arthritis, sacroiliitis, primary sclerosing cholangitis, pyoderma gangrenous, erythema nodosum, uveitis, episcleritis, osteopenia, and osteoporosis. Disease extension for UC was evaluated using the Montreal classification of proctitis (E1), left-sided colitis (E2), and extensive colitis (E3). For CD, the localization was classified as ileal, colonic, ileocolonic, upper gastrointestinal, or perianal involvement. The CD phenotype is divided into inflammatory, stricturing, penetrating, and perianal diseases. Acute and chronic complications related to this disease include toxic megacolon, perforation, colon cancer, massive hemorrhage, intestinal obstruction, abdominal sepsis, and pouchitis. Current treatment includes 5-aminosalicylates (5-ASA), local and systemic steroids, thiopurines, and biological treatment. Finally, the presence of thiopurine intolerance (presence of adverse effects with the use of short-term or long-term therapy that lead to discontinuation of treatment^[16]^) and resistance (presence of symptoms due to lack of levels of the drug or its metabolites in the blood^[17]^), steroid dependence (inability to remove steroids below the equivalent of prednisolone 10 mg/day or budesonide 3 mg/day within 3 months of starting steroids, relapse within 3 months of stopping steroids, or the need for more than a single course of corticosteroids in 1 year^[18]^), and resistance (inadequate or no response to steroid treatment after 40–60 mg prednisone or equivalent of oral steroids within 30 days and clinical improvement after treatment with high-dose intravenous steroids within 7–10 days^[19]^) were also evaluated.

### 2.1 Delay at diagnosis

Diagnostic delay was defined as >1 year from the onset of symptoms to a confirmed diagnosis of UC and >2 years for CD.^[20]^

### 2.2 Statistical analysis

Descriptive statistics were used, quantitative variables were expressed as mean ± standard deviation, and qualitative variables were expressed as frequency and percentage (%). We used the Student *t* test for continuous variables and the chi-square test for categorical variables. Multivariate analysis with logistic regression was performed to identify the confounding factors. Statistical analysis was performed using the SPSS v.24 statistical program (University of Chicago, Chicago Illinois, United States of America). Odds ratios (ORs) and 95% confidence intervals (CIs) were considered for the evaluation of association strength. Differences were considered statistically significant at *P* < .05. The power of the analysis was 62.8%.

## 3 Ethical considerations

This study was carried out following the guidelines of good clinical practice and was approved by the Central Research and Ethics Committee (approval number 2309 in Mexico City), and all centers were approved by the research and ethics committee prior to the recollection of clinical and demographic data.

## 4 Results

A total of 4714 patients with IBD from 9 Latin American and Caribbean countries were included in the present study. The number of patients included in each country was distributed as follows: 2289 from Colombia (48.5%), 73 from Cuba (1.5%), 354 from the Dominican Republic (7.5%), 309 from Ecuador (6.5%), 1304 from Mexico (27.6%), 32 from Peru (0.6%), 160 from Puerto Rico (3.3%), 23 from Uruguay (0.8%), and 170 from Venezuela (3.6%). Of the 4714 patients, 3374 (71.6%) had UC, 1291 (27.3%) had CD, and 49 (1.1%) had IC.

### 4.1 IBD frequency in Latin America and the Caribbean

CD was more frequent than UC in the following countries: Puerto Rico 71.9%, Dominican Republic 61.0%, and Peru 53.1%, while in the rest of the countries UC was most frequent: Colombia 78.6%, Venezuela 78.2%, Mexico 75.5%, Cuba 69.9%, Ecuador 64.1%, Uruguay 60.9% as shown in Table 1.

**Table 1:** Latin American and Caribbean countries in EPILATAM multicenter study.

Countries	IBD n = 4714	UC n = 3374	CD n = 1291	IC n = 49
Cuba (%)	73	51 (69.9)	22 (30.1)	0
Mexico (%)	1304	984 (75.5)	312 (23.9)	8 (0.6)
Peru (%)	32	15 (46.9)	17 (53.1)	0
Puerto Rico (%)	160	45 (28.1)	115 (71.9)	0
Dominican Republic (%)	354	134 (37.9)	216 (61.0)	4 (1.1)
Uruguay (%)	23	14 (60.9)	9 (39.1)	0
Venezuela (%)	170	133 (78.2)	36 (21.2)	1 (0.6)
Colombia (%)	2289	1800 (78.6)	469 (20.5)	20 (0.9)
Ecuador (%)	309	198 (64.1)	95 (30.7)	16 (5.2)

**Figure 1:**
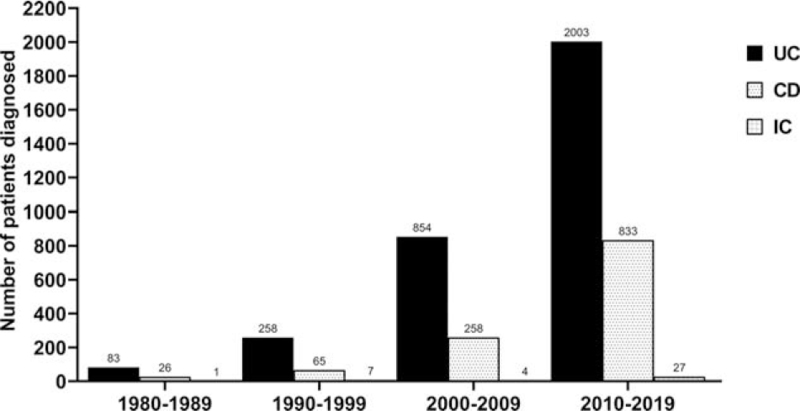
Increase in frequency of IBD diagnosis in the last decades. CD = Crohn disease, IBD = inflammatory bowel disease, IC = indeterminate colitis, UC = ulcerative colitis.

It is important to note that the frequency of IBD diagnosis has increased significantly in the last decade, with an increase of up to 7.7 times for UC, 12.8 times for CD, and 3.8 for IC, as shown in Fig. 1.

### 4.2 Demographic and clinical characteristics

Of the 4714 patients, 2942 (52.9%) were women and 2222 (47.1%) were men, with a median current age of 47 ± 16.95 years, and a mean disease duration of 8 years of disease duration (1–65 years). Of the 3374 patients with UC, the most frequent extension was extensive colitis (E3) in 1614 patients (47.8%), left-sided colitis (E2) in 961 patients (28.5%), and proctitis (E1) in 799 patients (23.7%). Of the 1291 patients with CD, the most frequent location was ileocolonic in 482 (37.3%) patients, ileal in 463 (35.9%), colonic in 226 (17.5%), perianal in 74 (5.7%), and upper gastrointestinal tract in 46 (3.6%). The most common CD phenotypes were inflammation (43.8%), stricturing (29.7%), penetrating (20.3%), and perianal disease (6.2%).

Smoking was present in 23% and 15.7% of the patients with UC and CD, respectively. Previous appendectomy was present in 11.7% and tonsillectomy in 7.5% of patients. The frequency of EIMs occurred in 36.7%, being the most frequent by order of frequency: arthralgia in 22.8%, ankylosing spondylitis in 13.1%, fatigue 12.61%, arthritis in 10.4%, uveitis in 2.9%, sacroiliitis in 2.5%, primary sclerosing in 1.9%, and pyoderma gangrenous in 1.4%.

### 4.3 Age at diagnosis of IBD

**Figure 2:**
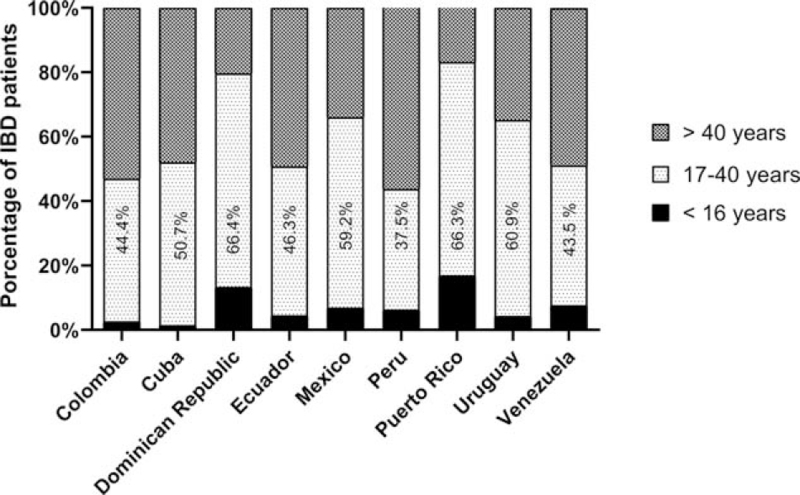
Age at diagnosis of IBD. IBD = inflammatory bowel disease.

The most frequent age at diagnosis of IBD is between 17 and 40 years, followed by those older than 40 years and younger than 16 years, as shown in Fig. 2.

### 4.4 Delay on IBD diagnosis

The age at onset of clinical symptoms was classified according to the decade in which it occurred; these groups were compared with the year of the confirmed diagnosis of IBD. A significant difference was observed in the last 4 decades comparing timely diagnosis and delay at diagnosis, for UC in 1980 to 1989 67.3% versus 32.4% (*P* = .009), in 1990 to 1999 66.7% versus 33.3% (*P* = .00003), in 2000 to 2009 78.1% versus 21.9% (*P* = .0003), and 2010 to 2019 90.7% versus 9.3% (*P* < .0001). For CD in 1980 to 1989 76.2% versus 23.8% (*P* = .25), in 1990 to 1999 64.5% versus 35.5% (*P* < .0001), in 2010 to 2019 91.7% versus 8.3% (*P* < .0001). The frequency of timely diagnosis has increased from 67.3% to 90.7% for UC and from 76.2% to 91.7% for CD in the last three decades.

### 4.5 Clinical characterization of IBD in Latin America and the Caribbean

To describe the clinical characteristics of patients with IBD, 2 study groups were compared: Caribbean countries, Cuba, Puerto Rico, and the Dominican Republic; and Latin American countries: Colombia, Ecuador, Mexico, Peru, Uruguay, and Venezuela. Several clinical differences were found between the Caribbean and Latin American countries (Table 2).

**Table 2:** Clinical differences of patients from Latin America and Caribbean.

	Caribbean	Latin America	*P* value	OR	CI 95%
Family history of IBD	14.0%	2.1%	**.0001**	7.71	5.18–11.47
Penetrating behavior in CD	34.8%	14.8%	**.0001**	3.17	2.40–4.20
Steroid dependence	12.9%	8.4%	**.001**	1.61	1.20–2.16
Steroid resistance	9.9%	0.7%	**.0001**	15.39	8.37–28.3
Thiopurine intolerance	6.1%	5.4%	.27	1.14	0.77–1.70
Pancolitis in UC	43.9%	48.1%	.12	0.84	0.64–1.10
Extraintestinal manifestations	59.1%	29.5%	**.0001**	3.44	2.84–4.18
IBD surgery	31.2%	7.3%	**.0001**	4.51	3.68–5.54
IBD hospitalizations	72.7%	48.1%	**.001**	2.87	2.37–3.48

The main and significant clinical characteristics between Caribbean and Latin America both regions were family history of IBD (14.0% vs 2.1%, *P* = .0001, OR 7.71, CI 95% 5.18–11.47); penetrating behavior in CD (34.8% vs 14.8%, *P* = .0001, OR 3.17, CI 95% 2.40–4.20); steroid dependence (12.9% vs 8.4%, *P* = .001, OR 1.61, CI 95% 1.20–2.16); steroid resistance (9.9% vs 0.7%, *P* = .0001, OR 1.61, CI 95% 8.37–28.3); EIMs (59.1% vs 29.5%, *P* = .0001, OR 3.44, CI 2.84–4.18); IBD surgery (31.2% vs 7.3%, *P* = .0001, OR 4.51, CI 95% 3.68–5.54), IBD hospitalizations (72.7% vs 48.1%, *P* = .0001, OR 2.87, CI 95% 2.37–3.48).

### 4.6 Medical treatment

The current treatment for IBD was based on 71.0% 5-ASA (mesalazine 96.3%, sulfasalazine3.7%), 27.4% steroids (oral steroids 96.4%, budesonide 3.6%), 27.4% thiopurines (azathioprine 96.4%, 6-mercaptopurine 0.6%; methotrexate, 3.0%), 23.1% biological therapy, and 0.1% small molecule inhibitors. Of the 1092 patients treated with biological therapy, 68.7% were treated with infliximab, 23.2% with adalimumab, 3.2% with vedolizumab, 2.9% with golimumab, 1.3% with ustekinumab, and 0.4% with certolizumab pegol. The use of small-molecule inhibitors such as tofacitinib was 0.1%. Differences in the frequency of medical treatment were found between the Caribbean and Latin American countries, with a high frequency of use of 5-ASA and steroids in Latin America compared with Caribbean countries, where the most frequent therapy was biological treatment, as shown in Table 3. In UC, the use of biological therapy was 36.8% versus 3.64 (*P* < .0001) and for CD, 66.8% versus 12.8 (*P* < .0001) for the Caribbean and Latin America, respectively.

**Table 3:** Current IBD treatment in Latin America and the Caribbean.

Treatment	Caribbean	Latin America	*P* value	OR	CI 95%
5-ASA	32.5%	76.5%	**<.0001**	0.14	0.12–0.17
Steroids	10.1%	31.4%	**<.0001**	0.24	0.18–0.31
Thiopurines	25.9%	27.6%	.20	0.91	0.75–1.14
Biological treatment	56.4%	18.4%	**<.0001**	5.71	4.77–6.85

### 4.7 Factors associated with the use of biological therapy in Latin America and the Caribbean

Univariate and multivariate analyses showed that factors associated with the use of biological therapy were extensive colitis for UC and penetrating behavior in CD, and steroid resistance, steroid dependence, presence of EIMs, and IBD-related surgeries were associated with IBD (Table 4).

**Table 4:** Factors associated with the use of biological therapy in IBD.

	Univariate analysis			Multivariate analysis		
	*P* value	OR	CI 95%	*P* value	OR	CI 95%
Extensive colitis in UC	<.0001	1.65	1.36–2.0	**<.0001**	1.605	1.25–1.87
Penetrating behavior in CD	<.0001	2.52	1.91–3.32	**<.0001**	2.219	1.75–3.12
Steroid dependence	<.0001	5.2	3.2–8.46	**<.0001**	4.501	3.0–7.43
Steroid resistance	<.0001	1.96	1.47–2.61	**.001**	1.823	1.28–2.45
Thiopurine intolerance	.02	1.47	1.01–2.14	.609	.889	0.644–1.23
Extraintestinal Manifestations	<.0001	1.98	1.64–2.40	**<.0001**	1.812	1.45–2.04
IBD surgery	<.0001	3.59	2.99–4.31	**<.0001**	3.59	2.98–4.30

## 5 Discussion

This retrospective cohort study demonstrated an important increase in the diagnosis of IBD in the last decade, as well as differential clinical characterization of IBD between Latin America and Caribbean countries.

IBD is considered a disease that only affects Caucasian or white populations, located mainly in Europe, North America, and Australia,^[6,21]^ and is associated with morbidity, mortality, and substantial costs to the healthcare system.^[22,23]^

The findings of the present study have shown that CD had a higher frequency of 53.1% compared with a previous study that reported a low frequency of CD in Puerto Rico in 2009.^[11]^ Subsequently, the frequency of CD increased from 47.1% to 52.9% for UC.^[12,13,24,25]^ In the Dominican Republic, CD predominated at 61.0%; in Peru, CD had a frequency of 53.1%, which increased from a previously published study that showed a frequency of 23% for CD and 77% for UC.^[26]^ UC was predominant in Venezuela (78.2%), Cuba (69.9%), and Mexico (78.6%). This is consistent with a previous study where the Hispanic population had a higher frequency of UC than CD^[12,26]^ compared with African Americans and Caucasians, in whom CD is more frequent.^[27,28]^

It is important to note that comparing the clinical characteristics between both regions, Caribbean versus Latin America, there is a more aggressive clinical course in IBD patients from the Caribbean countries with a greater number of hospitalizations related to IBD, presence of EIMs, penetrating behavior in CD, IBD-related surgeries, steroid resistance, and steroid dependence. Higher hospitalization rates have been observed in North America and Europe than in Asia and Latin America.^[5,29]^ The frequency of extensive colitis or pancolitis in our study was 48.1% in Latin American countries compared with the Caribbean (43.9%), which is in accordance with the Caucasian population.^[30]^ These findings support the notion that IBD is a heterogeneous entity in both Latin America and the Caribbean. Environmental factors, such as socioeconomic status, exposure to infections, abuse of antibiotics, and poor hygiene, might help explain the epidemiological differences between populations.^[31,32]^

In the present study, there was a significantly higher use of biological therapy in the Caribbean countries than in Latin America, which is in accordance with previous studies, where the use of biological therapy was similar in the Caribbean and Caucasian countries, suggesting that in this region, the disease behavior is more aggressive than in Latin American countries. Based on the genetic history of these regions, Latin American countries show a primarily Native American ancestry, followed by European descent and a minimal proportion of African descent, contrary to what happens in the Caribbean regions, where the first pulse of European migration has been demonstrated in genetic studies followed by multiple pulses of African descent that contributed significantly to genetic ancestry in the Caribbean.^[33]^

We determined that the factors associated with the use of biological therapy were extensive colitis or pancolitis in UC, penetrating behavior in CD, steroid dependence, steroid resistance, presence of EIMs, and IBD-related surgeries in IBD, which translates into a more complicated disease and is refractory to conventional treatment, which is one of the main indications for starting biological therapy.

The main advantage of the present study is that it was conducted in several countries from Latin America and the Caribbean, which characterizes a cohort of patients from these 2 regions clinically and epidemiologically, highlighting important differences in IBD and the medical treatment used. This study has some limitations; for example, all patients were recruited from IBD specialized medical centers, other countries are missing mainly from Central and South America, and a limited number of IBD patients were included from some countries.

This multicenter cohort study was carried out in several countries in Latin America and the Caribbean, which demonstrated that the main and significant clinical characteristics between the Caribbean and Latin America were a family history of IBD, penetrating behavior in CD, steroid dependence, steroid resistance, EIMs, IBD surgery, IBD hospitalizations, and the factors associated with the use of biological therapy were extensive colitis for UC and penetrating behavior in CD as well as steroid resistance, steroid dependence, presence of EIMs, and IBD-related surgeries in IBD patients (Supplemental Digital Content).

## Author contributions

**Conceptualization:** Jesus K. Yamamoto-Furusho.

**Data curation:** Jesus K. Yamamoto-Furusho, Norma N. Parra-Holguín, Fabian Juliao-Baños, Fabian Puentes, Rocio Lopez, Francisco Bosques-Padilla, Esther A. Torres, Humberto Nieves-Jimenez, Guillermo Veitia-Velasquez, Maria L. Jara-Alba, Socrates Bautista, Felipe N. Piñol-Jimenez, Pablo Salgado-Rosado, Keyla C. Villa-Ovalles, Yudelka A. Abreu-Martinez, Zunilda Borges, Santiago Davila-Bedoya, Guillermo Otoya-Moreno, Beatriz Iade-Vergara.

**Formal analysis:** Jesus K. Yamamoto-Furusho, Norma N. Parra-Holguín.

**Methodology:** Jesus K. Yamamoto-Furusho, EPILATAM Study Group.

**Project administration:** EPILATAM Study Group.

**Resources:** EPILATAM Study Group.

**Software:** EPILATAM Study Group.

**Supervision:** Jesus K. Yamamoto-Furusho.

**Validation:** Jesus K. Yamamoto-Furusho, Fabian Juliao-Baños, Fabian Puentes, Rocio Lopez, Francisco Bosques-Padilla, Esther A. Torres, Humberto Nieves-Jimenez, Guillermo Veitia-Velasquez, Maria L. Jara-Alba, Socrates Bautista, Felipe N. Piñol-Jimenez, Pablo Salgado-Rosado, Keyla C. Villa-Ovalles, Yudelka A. Abreu-Martinez, Zunilda Borges, Santiago Davila-Bedoya, Guillermo Otoya-Moreno, Beatriz Iade-Vergara.

**Visualization:** Jesus K. Yamamoto-Furusho, Fabian Juliao-Baños.

**Writing – original draft:** Jesus K. Yamamoto-Furusho, Norma N. Parra-Holguín.

**Writing – review & editing:** Jesus K. Yamamoto-Furusho, Fabian Juliao-Baños, Fabian Puentes, Rocio Lopez, Francisco Bosques-Padilla, Esther A. Torres, Humberto Nieves-Jimenez, Guillermo Veitia-Velasquez, Maria L. Jara-Alba, Socrates Bautista, Felipe N. Piñol-Jimenez, Pablo Salgado-Rosado, Keyla C. Villa-Ovalles, Yudelka A. Abreu-Martinez, Zunilda Borges, Santiago Davila-Bedoya, Guillermo Otoya-Moreno, Beatriz Iade-Vergara.

## Supplementary Material

Supplemental Digital Content
