# Supplementary material for: Clinical differentiation of inflammatory bowel disease (IBD) in Latin America and the Caribbean
Source: Medicine (Baltimore). 2022 Jan 21;101(3):e28624. doi: 10.1097/MD.0000000000028624 (PMC8772634; doi:10.1097/MD.0000000000028624)
Supplement: Supplemental Digital Content [file medi-101-e28624-s001.docx]

**Authors of EPILATAM Study Group
Supplement 1**

**MEXICO**

1. **Jesús Kazuo Yamamoto Furusho**

Inflammatory Bowel Disease Clinic, Gastroenterology Department, National Institute of Medical Science and Nutrition Salvador Zubirán, Mexico City, Mexico.

1. **Norma Nathaly Parra Holguín**

Inflammatory Bowel Disease Clinic, Gastroenterology Department, National Institute of Medical Science and Nutrition Salvador Zubirán, Mexico City, Mexico.

1. **Francisco Bosques Padilla**

Departamento de Gastroenterología Hospital Universitario Dr.José Eleuterio González, Universidad Autónoma de Nuevo León, Monterrey, Nuevo León, Mexico.

**COLOMBIA**

1. **Fabián Juliao Baños**

Hospital Pablo Tobón Uribe, Medellín, Colombia.

1. **Mateo Arrubla**

Hospital Pablo Tobón Uribe, Medellín, Colombia.

1. **Joselyn Camargo**

Hospital Pablo Tobón Uribe, Medellín, Colombia.

1. **Fabián Puentes**

Cirujanos Unidos. Manizales, Colombia.

1. **Lázaro Arango**

Cirujanos Unidos. Manizales, Colombia.

1. **Rocío López**

Fundación Santa Fe, Bogotá, Colombia.

1. **Rafael García**

Fundación Santa Fe, Bogotá, Colombia.

1. **Belén Mendoza**

Fundación Santa Fe, Bogotá, Colombia.

1. **María A. Saffon**

Instituto Gastroclínico, Medellín, Colombia.

1. **Luis F. Roldan**

Instituto Gastroclínico. Medellín, Colombia.

1. **Julio Zuleta**

Instituto Gastroclínico. Medellín, Colombia.

1. **Gustavo Reyes**

Clínica Colombia, Bogotá, Colombia.

1. **Viviana Parra**

Gastroadvanced, Bogotá-Medellín, Colombia.

1. **Cristian Flórez**

Gastroadvanced, Bogotá-Medellín, Colombia.

1. **Edilberto Nuñez**

Gastroadvanced, Bogotá-Medellín, Colombia.

1. **María T. Galiano**

MTG Servimed SAS, Bogotá, Colombia.

1. **Marcos Barraza**

Dr. Endodigestivos. Pereira, Colombia.

1. **Isabel C. Sanchez**

Dr. Endodigestivos, Pereira, Colombia

1. **Jenny L. Molano**

Emdiagnóstica SAS, Bogotá, Colombia

1. **Jorge I. Lizarazo**

Emdiagnóstica SAS, Bogotá, Colombia.

1. **Iván Cuellar**

Emdiagnóstica SAS, Bogotá, Colombia.

1. **Eligio Álvarez**

IMAT, Montería. Colombia.

1. **Rubén Corrales**

Clínica Intermedios. Montería, Colombia.

1. **Fabio Gil**

Clínica Colombia, Bogotá, Colombia.

1. **Luz E. Vargas**

Clínica La Misericordia, Barranquilla, Colombia.

1. **Patricia Álvarez**

Clínica La Carolina, Bogotá, Colombia.

1. **Luis M. Limas**

LIMEQ. Tunja, Colombia.

1. **Paola Yance**

Gastrosalud, Santa Marta, Colombia.

1. **Robín Prieto**

Hospital Central de la Policía, Bogotá, Colombia.

1. **Hernán Ballén**

Hospital Central de la Policía, Bogotá, Colombia.

1. **Lidsay Delgado**

Hospital Central de la Policía, Bogotá, Colombia.

**PUERTO RICO**

1. **Esther A. Torres**

Universidad de Puerto Rico, San Juan, Puerto Rico.

1. **Humberto Nieves Jimenéz**

Universidad de Puerto Rico, San Juan, Puerto Rico.

**VENEZUELA**

1. **Guillermo Rafael Veitia Velásquez**

Servicio de Gastroenterología del Hospital Vargas de Caracas, Caracas, Venezuela.

**DOMINICAN REPUBLIC**

1. **Sócrates Bautista**

CEDIMAT Centro de Gastroenterología, República Dominicana

1. **Keyla Cristina Villa Ovalles**

CEDIMAT Centro de Gastroenterología, República Dominicana

1. **Yudelka Altragracias Abreu Martínez**

Hospital Regional Universitario José María Cabral y Baez, Santiago, República Dominicana

1. **Zunilda Borges**

Hospital Regional Universitario José María Cabral y Baez, Santiago, República Dominicana

1. **Aleydi Miosotis Frías Santana**

Unidad de Enfermedad Inflamatoria Intestinal Hospital Moscoso Puello

1. **Gianna Blanco**

Unidad de Enfermedad Inflamatoria Intestinal Hospital Moscoso Puello

1. **Ivelisse Liliana Jiménez**

CEDIMAT Centro de Gastroenterología, República Dominicana

1. **Clarali Almonte Nuñez**

Hospital Padre Billini, República Dominicana

1. **Miosotis Elizabeth Suarez**

Hospital Padre Billini, República Dominicana

**CUBA**

1. **Felipe Neri Piñol Jimenez**

Centro Nacional de Cirugía de Mínimo Acceso, Habana, Cuba

**PERU**

1. **Guillermo Otoya Moreno**

Consultorio Privado, Lima, Perú.

**ECUADOR**

1. **María Luisa Jara Alba**

Hospital Dr. Teodoro Maldonado Carbo - IESS, Guayaquil, Ecuador.

1. **Pablo Salgado Rosado**

Hospital Dr. Teodoro Maldonado Carbo - IESS, Guayaquil, Ecuador.

1. **Dr.Santiago Davila Bedoya**

Hospital Carlos Andrade Marin, Quito, Ecuador

1. **Dr. David Andrade Zamora**

Hospital de Especialidades José Carrasco Arteaga – Instituto Ecuatoriano de Seguridad Social. Cuenca, Ecuador

1. **Dr. Rubén Gustavo Muñoz Cedeño**

Servicio de Gastroenterología del Hospital de Especialidades Guayaquil Dr. Abel Gilbert,

1. **Dra. Vielka Beatriz Cedeño Arauz**

Hospital del Instituto de Seguridad Social (IESS), Portoviejo, Manabí, Ecuador **URUGUAY**

1. **Beatriz Iadé Vergara**

CASMU COSEM Uruguay
